# Doping Free and Amorphous NiO_x_ Film via UV Irradiation for Efficient Inverted Perovskite Solar Cells

**DOI:** 10.1002/advs.202201543

**Published:** 2022-04-25

**Authors:** Qing Lian, Peng‐lai Wang, Guoliang Wang, Xian Zhang, Yulan Huang, Dongyang Li, Guojun Mi, Run Shi, Abbas Amini, Liang Zhang, Chun Cheng

**Affiliations:** ^1^ Department of Materials Science and Engineering Southern University of Science and Technology Shenzhen Guangdong Province 518055 China; ^2^ School of Chemistry and Molecular Engineering East China Normal University Shanghai 200062 China; ^3^ Center for Infrastructure Engineering Western Sydney University Kingswood NSW 2751 Australia

**Keywords:** green synthesis, inverted perovskite solar cells, NiO_x_, photochemistry synthesis, UV irradiation

## Abstract

High crystallization and conductivity are always required for inorganic carrier transport materials for cheap and high‐performance inverted perovskite solar cells (PSCs). High temperature and external doping are inevitably introduced and thus greatly hamper the applications of inorganic materials for mass production of flexible and tandem devices. Here, an amorphous and dopant‐free inorganic material, Ni^3+^‐rich NiO_x_, is reported to be fabricated by a novel UV irradiation strategy, which is facile, easily scaled‐up, and energy‐saving because all the processing temperatures are below 82 ℃. The as‐prepared NiO_x_ film shows highly improved conductivity and hole extraction ability. The rigid and flexible PSCs present the champion efficiencies of 22.45% and 19.7%, respectively. This work fills the gap of preparing metal oxide films at the temperature below 150 °C for inverted PSCs with the high efficiency of >22%. More importantly, this work upgrades the substantial understanding about inorganic materials to function well as efficient carrier transport layers without external doping and high crystallization.

## Graphical abstract

Fabrication of effective inorganic hole transport films at low temperature is crucial to move perovskite solar cells one step closer to mass production and then commercialization. Here, the authors report a photochemistry method to synthesize NiO_x_ hole transport layer for perovskite solar cells with a champion performance of 22.45%.

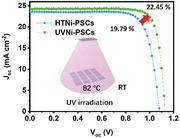

## 1 Introduction

Despite rapid lab‐on‐the‐chip breakthroughs in hybrid organic–inorganic, there are serious challenges appearing against the commercialization and mass production of halide perovskite solar cells (PSCs), including the lack of facile and low temperature protocols.^[^ ^1^ ^]^ Inverted planar devices, compared to the regular ones, have received specific attentions for their simple architecture and fabrication process; they are useful for large‐area flexible devices, and excellently compatible with tandem devices. In inverted PSCs, inorganic hole transport materials demonstrate admiring advantages such as higher carrier mobility, superior stability, and low‐cost, and thus have been intensively investigated.^[^ ^2^ ^]^ Among them, NiO_x_ has recently been emerged because of its adapted valence band to that of perovskite, ensuring a great potential for high‐performance inverted PSCs.^[^ ^3^ ^]^

The reported NiO_x_ hole transport layers (HTLs) are always a mixture of nickel oxides with dominant NiO composition. However, stoichiometric NiO is a wide‐bandgap semiconductor with poor conductivity,^[^ ^4^ ^]^ that severely inhibits hole transport. To address this problem, various strategies have been developed such as inorganic elements^[^ ^4^, ^5^ ^]^ and small molecules doping,^[^ ^6^ ^]^ and post‐treatments^[^ ^7^ ^]^ including sulphuration, annealing, etc.^[^ ^5^, ^7^, ^8^ ^]^ Upon these efforts, the highest power conversion efficiency (PCE) of NiO_x_ nanocrystal‐based PSCs has reached 22.74%.^[^ ^9^ ^]^ These achievements suggest the superior potential of NiO_x_ film for efficient inverted PSCs. Currently, two mainstream approaches, nanocrystals ink^[^ ^6b^ ^]^ and sol–gel method,^[^ ^6c^ ^]^ are under development for the preparation of crystalline NiO_x_ films. High‐temperature processes (>200 °C) are always needed to improve the crystallization of NiO_x_ and doping effect for enhanced hole extraction and transport ability of NiO_x_ as well as boosted PCE of PSCs. Additionally, the preparation of NiO_x_ nanocrystals ink has a complex procedure (centrifugation, vacuum drying, redispersing, etc.).^[^ ^8^, ^10^ ^]^ These obstacles hamper the mass fabrication and promising applications of NiO_x_ film for flexible devices. Therefore, it is yet a crucial challenge to facilely prepare effective NiO_x_ HTLs at temperatures <150 °C.

Here, we report a strategy that simultaneously addresses conductivity, high temperature, complex process, and uniformity issues that are facing current NiO_x_‐based efficient PSCs embodiments. We found the amorphous, Ni^3+^‐rich, and uniform NiO_x_ film with a large area by using UV irradiation method at 82 °C. The remarkable increase in the conductivity enabled the doping‐free NiO_x_ film to be used for effective hole extraction. This led to a great improvement in fill factor and boosted PCEs (22.45% and 19.70% for rigid and flexible devices, respectively) for the NiO_x_‐based inverted PSCs. Our work fills the gap of preparing metal oxide film at the temperature under 150 °C for efficient PSCs, with the great potential for the mass production of PSCs.

## 2 Results and Discussion

### 2.1 Fabrication and Characterization of UV‐NiO_x_ Film

**Figure 1:**
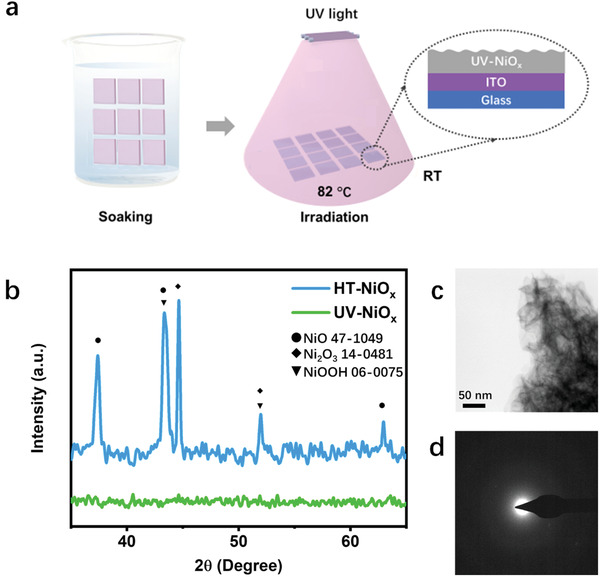
Fabrication and structure characterizations of UV‐NiO_x_ film. a) Schematic of the preparation procedure of UV‐NiO_x_ film. b) XRD patterns of HT and UV‐NiO_x_ films. c) STEM‐DF2 image, and d) SAED taken from UV‐NiO_x_ sample. The UV‐NiO_x_ has an amorphous structure according to the above XRD and SAED results. Scale bar is 50 nm.

The preparation procedure of NiO_x_ films by UV irradiation (UV‐NiO_x_) is shown in **Figure** **1**a. In brief, a batch of ITO substrates was soaked in Ni^2+^ solution for tens of seconds, then transferred to the commercial UV box after drying. UV‐NiO_x_ film was obtained after the UV irradiation in ambient conditions for several minutes. Notably, the surface temperature of ITO substrate increased upon UV irradiation and stabilized quickly at 82 ℃. For comparison, the control HT‐NiO_x_ film was prepared by the conventional high‐temperature annealing of sol–gel Ni(acac)_2_ (see details in Experimental Section).^[^ ^6c^ ^]^

X‐ray diffraction (XRD) and scanning transmission electron microscopy (STEM) were used to investigate the crystallinity of NiO_x_ samples. We identified the HT‐NiO_x_ film with a mixture of crystalline NiO, Ni_2_O_3_, and NiOOH while UV‐NiO_x_ film possessed an amorphous structure (Figure 1b). Further STEM and selected area electron diffraction (SAED) results confirmed the amorphous features of UV‐NiO_x_ film with irregular morphology and diffuse halo (Figure 1c,d).^[^ ^11^ ^]^ In contrast, HT‐NiO_x_ possessed a nanoparticle morphology and poly‐crystalline structure (Figure S1, Supporting Information). The low process temperature (≈82 °C) and short time irradiation (several minutes) during the film preparation were the rationale for the formation of amorphous structure of UV‐NiO_x_ film; this can provide uniformly physical and chemical properties for large scale geometries.

**Figure 2:**
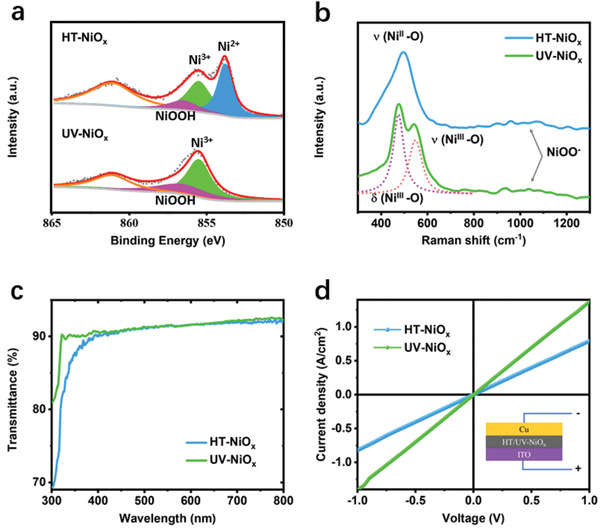
NiO_x_ film components, optical and electrical characterizations, and the uniformity. a) High‐resolution Ni2p_3/2_ XPS spectrum. XPS peaks of Ni^2+^ and Ni^3+^ composites are observed in HT‐NiO_x_, while no Ni^3+^ peak is observed in UV‐NiO_x_. b) Raman spectra and c) transmittance spectra of HT and UV‐NiO_x_ film. d) Dark current−voltage curves of hole‐only devices. UV‐NiO_x_ film exhibits higher current density at the same forward bias than HT‐NiO_x_ film, which indicates better hole extraction capability.

We probe the compositions of NiO_x_ films by using X‐ray photoelectron spectroscopy (XPS) and Raman spectroscopy. From **Figure** **2**a, HT‐NiO_x_ film is identified as a mixture of Ni^2+^ and Ni^3+^ composites, including NiO, Ni_2_O_3_, and NiOOH (64.9%:16.5%:18.6% in molar ratio), where Ni^2+^ is rich with the molar ratio of Ni^2+^:Ni^3+^ as 64.9%:35.1%. Notably, UV‐NiO_x_ film consists of almost 100% Ni^3+^ composition; the molar ratio of Ni^3+^ for Ni_2_O_3_ and Ni^3+^ for NiOOH is 69.3%:30.7% (Figure 2a). This indicates that UV irradiation can effectively convert Ni^2+^ to Ni^3+^ at a relatively low temperature within a short period of time. Further, the Raman spectroscopy is used to distinguish Ni‐O bands as shown in Figure 2b. We observe the peak for A_1g_ stretching mode (*ν*(Ni^II^‐O)) of dehydrated form of nickel hydroxide in HT‐NiO_x_ film, and the peaks for *E*_g_ bending vibration (*δ*(Ni^III^‐O)) and A_1g_ stretching vibration (*ν*(Ni^III^‐O)) modes of Ni^III^‐O in dehydrated form of nickel oxyhydroxide (NiOOH) in UV‐NiO_x_.^[^ ^12^ ^]^ The peaks attributed to NiOO‐ are also observed in both HT‐ and UV‐NiO_x_.^[^ ^12^ ^]^ More details of Raman results are provided in Discussion S1 and Figure S2 (Supporting Information). All the above compositional characterizations show that UV‐NiO_x_ film is Ni^3+^‐dominated while HT‐NiO_x_ film is rich in Ni^2+^ components.

The optical transmittance of NiO_x_ films is further studied through UV–vis spectra. Both HT and UV‐NiO_x_ films exhibit high transmittance in the visible spectrum with an average transmittance >90% (Figure 2c). Specifically, UV‐NiO_x_ film has a higher transmittance than HT‐NiO_x_ film at the wavelength between 300 and 400 nm. The high transparency of UV‐NiO_x_ film minimizes optical losses of PSCs, ensuring the higher photocurrent of PSCs. We further study and confirm the uniformity of UV‐NiO_x_ film on the compositions and low sheet resistance (Figure S2e, Supporting Information). The excellent conductivity of UV‐NiO_x_ film can be well understood by knowing that Ni^3+^ donates more vacancies that improve the electrical conductivity.^[^ ^2^, ^4^, ^13^ ^]^ To study the hole extraction ability of NiO_x_ films, the dark current–voltage curves of hole‐only devices of ITO/NiO_x_/Cu are obtained (Figure 2d). UV‐NiO_x_ film exhibits higher current density at the same forward bias than HT‐NiO_x_ film, which indicates its better hole extraction capability.^[^ ^5a^ ^]^ Consequently, the conductive UV‐NiO_x_ film boosts the hole transport from perovskite to HTL layers, and thus the NiO_x_‐based PSCs have a potential to achieve higher PCE by replacing HT‐NiO_x_ with UV‐NiO_x_. From the above comprehensive characterizations, it is concluded that the as‐prepared UV‐NiO_x_ film is a promising HTL with high optical transmittance, enhanced conductivity, and hole extract ability, very uniform composition, and electrical properties; these characteristics endow UV‐NiO_x_ film with a great potential for mass manufacturing of effective PSCs.

**Figure 3:**
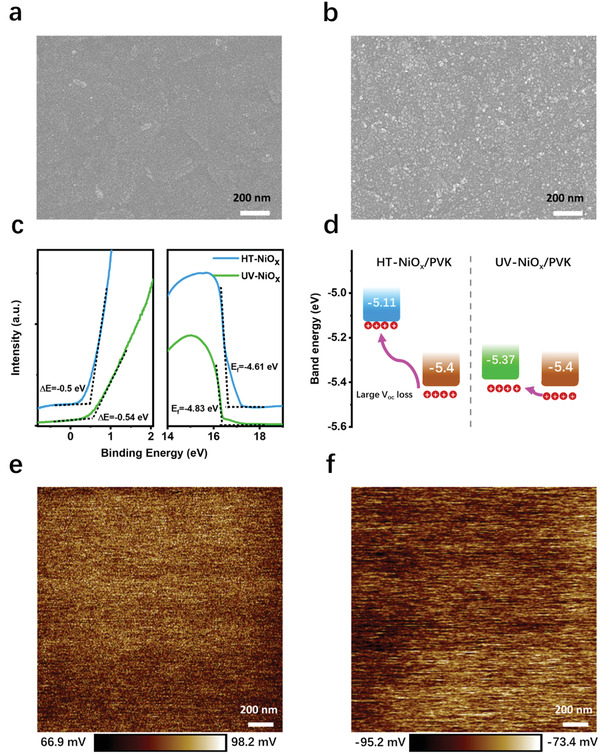
Surface properties of NiO_x_ film. Top‐view SEM images of a) HT and b) UV‐NiO_x_ films. c) UPS spectra of HT and UV‐NiO_x_ films. d) Schematic diagram of energy levels for different NiO_x_ and perovskite layers, the VBMs of HT‐/UV‐NiOx films are calculated from UPS as −5.11 and −5.37 eV, respectively. 2 × 2 µm KPFM images of e) HT‐NiO_x_ and f) UV‐NiO_x_ film. A lower average voltage is observed for UV‐NiO_x_ film, which indicates a higher work function than that of HT‐NiO_x_. Scale bar is 200 nm.

Scanning electron microscopy (SEM) and atomic force microscopy (AFM) are used to probe the surface morphology and height difference properties of NiO_x_ films. SEM images show a smooth surface for HT‐NiO_x_ film (**Figure** **3**a) and a relatively rough surface for UV‐NiO_x_ film with many spread white particles (Figure 3b). These dots are attributed to slightly self‐aggregated amorphous UV‐NiO_x_. Further, AFM images in Figure S3a,b (Supporting Information) reveal that *R*_qs_ of HT and UV‐NiO_x_ films are as low as 1.84 and 3.21 nm, respectively. Based on the SEM and AFM results, it is concluded that UV‐NiO_x_ film has a slightly rougher surface than that of HT‐NiO_x_ film that may suggest the better wetting property of UV‐NiO_x_ film.^[^ ^14^ ^]^ This is proved by the results from contact angle measurement (Figure S3c,d, Supporting Information), where HT‐NiO_x_ film has a reduced contact angle of 24.7°, smaller than that of UV‐NiO_x_ film (33.1°).

Ultraviolet photoelectron spectroscopy (UPS) was carried out to identify the energy levels of NiO_x_ films. From Figure 3c, the valence band maximum (VBM) of HT‐/UV‐NiO_x_ films were calculated as −5.11 and −5.37 eV, respectively. The scheme of energy‐level alignment for NiO_x_ and perovskite films was consequently constructed (Figure 3d). It is known that a better band alignment with the perovskite layer can reduce the deficiency of carrier transport between perovskite layer and NiO_x_ layer.^[^ ^7a,b^ ^]^ Here, the VBM difference between NiO_x_ film and perovskite shrinks to only 0.03 from 0.29 eV upon replacing HT‐NiO_x_ with UV‐NiO_x_; this setup can promote the extraction of holes from perovskite to NiO_x_ layer, while reducing the energy loss during the hole transfer.^[^ ^5a^ ^]^ In this way, the efficient holes extraction from perovskite to UV‐NiO_x_ film can be greatly promoted, and nonradiative recombination can be suppressed at the interface of UV‐NiO_x_/perovskite.

Kelvin probe force microscopy (KPFM) is used to measure the surface potential/work function of thin films by which the fluctuation can reveal the local distribution of surface traps.^[^ ^5a^ ^]^ Surface traps can bring self‐doping and thus induce local variation in the band bending and surface potential; these always cause negative impacts on the overall carrier transport. Figure 3e,f provides the KPFM results of NiO_x_ films; UV‐NiO_x_ film exhibits lower magnitudes for surface potential (Maximum: −74.3 mV, Minimum: −95.2 mV, and Mean: −80.1 mV) than those for HT‐NiO_x_ film (Maximum: 98.2 mV, Minimum: 66.9 mV, and Mean: 82.2 mV); this result suggests that the work function of UV‐NiO_x_ film is substantially reduced. The difference of mean values gives the change of work functions of these two films (162.3 mV), which is quite consistent with the result from UPS tests (220 mV). From the difference of maximum and minimum values, it is found that the surface potential of UV‐NiO_x_ film is more even across the surface than that of HT‐NiO_x_ film; this property is beneficial for carrier transport. This observation further confirms the fact that UV‐NiO_x_ film has much more uniform properties than HT‐NiO_x_ film, as revealed in Figure S2 (Supporting Information).

### 2.2 Proposed Reaction Mechanisms of NiO_x_ Films

In order to understand the reaction mechanism, comprehensive characterizations were carried out on the samples of UV‐NiO_x_ film prepared at different UV irradiation time. It was found that the morphology (SEM and TEM), crystal structure (XRD), and surface properties (Contact Angle measurement) of UV‐NiO_x_ films did not undergo evident changes. However, Raman data clearly showed a significant composition change of UV‐NiO_x_ film with the illumination time (Figure S4) that reveals the reaction process of NiO_x_. According to the reaction conditions and characterization results of the preparation of HT‐ and UV‐NiO_x_ films, the relevant reactions are proposed as follow:

(1) $$\begin{array}{ccc} & & \mathrm{Ni}{\left(\mathrm{acac}\right)}_{2}\left(s\right)\;+O_{2}\left(g\right)\overset{\mathrm{HT}\;=\;400{\;}^{\circ }C}{\to }\;\mathrm{aNiO}\;\mathrm{bN}i_{2}O_{3}\;\mathrm{cNiOOH}\left(s\right)\\ & & \;+H_{2}O\left(g\right)+CO_{2}\left(g\right) \end{array}$$

(2) $$\mathrm{Ni}{\left(\mathrm{OH}\right)}_{2}\left(s\right)\;+\;O_{2}\left(g\right)\overset{\mathrm{UV}}{\to }\mathrm{aN}i_{2}O_{3}\;\mathrm{bNiOOH}\left(s\right)\;+\;H_{2}O\left(g\right)$$

As clearly stated before, for the preparation of HT‐NiO_x_, Ni(acac)_2_ converts to Ni^2+^‐rich oxides by the pyrolysis and oxidation reaction of (1).^[^ ^6^, ^15^ ^]^ For the preparation of UV‐NiO_x_, the photon energy of 185 nm that is dominant in the spectrum of UV lamp emission (Figure S4a, Supporting Information), is calculated as 646.63 kJ mol^–1^; this is high enough to decompose Ni(OH)_2_ by breaking Ni—O and O—H bonds to form free radicals of Ni· and O· and H· (Table S1, Supporting Information).^[^ ^16^ ^]^ O_3_ generated by the UV irradiation of O_2_ in air (Equation S4, Supporting Information) has an extremely high oxidizing activity and thus it reacts with these free radicals to form Ni^3+^ compositions of NiOOH and Ni_2_O_3_ (Figure 2a,b) at a relatively low temperature of ≈82 ℃ (Figure S4b, Supporting Information). The low temperature also inhibits the crystallization of the precursor, forming an amorphous structure of UV‐NiO_x_ (Figure 1b–d) (Reaction (2), more details on the reaction mechanism are shown in Discussion S2 and Figure S4, Supporting Information).

### 2.3 Characterizations of Perovskite/NiO_x_ Films and Their Interfaces

Perovskite films were prepared over NiO_x_ layers to study the impacts of different NiO_x_ films on the morphology and photoelectric property of perovskite as well as their interface structure and properties. The top view SEM images of perovskite films on HT and UV‐NiO_x_ layers reveal that both perovskite films are well‐crystallized and pinhole‐free with the grain size of 400–800 nm (Figure S5a,b, Supporting Information) and similar XRD patterns (Figure S5c, Supporting Information). This suggests their identical morphology and structure, although the underneath NiO_x_ films have distinct component and properties (Figures 2, 3). This result is consistent with the cross‐section HAADF‐STEM images of NiO_x_‐based PSCs, where NiO_x_ and perovskite layers are almost identical in morphology and component distribution with the compact and smooth interfaces (Figure S6, Supporting Information).

**Figure 4:**
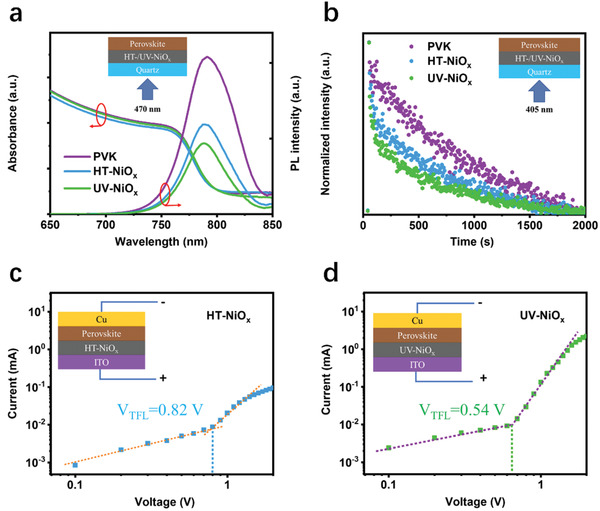
Structure and interface carrier transport properties of perovskite and NiO_x_ layers. a) Light absorption and PL emission of perovskite films on quartz, HT, and UV‐NiO_x_ films, and b) TRPL of perovskite films on quartz, HT, and UV‐NiO_x_ films. To understand the optoelectronic properties at the interfaces of NiO_x_/perovskite, the perovskite layer was excited from quartz/NiO_x_ side. Dark *J–V* characteristics of trap density and carrier mobility of the perovskite films are obtained for c) HT‐NiO_x_/ITO and d) UV‐NiO_x_/ITO with the device structure of ITO/HT‐ or UV‐NiO_x_/perovskite/Cu.

**Figure** **4**a shows the UV–vis absorption spectra of three perovskite films on quartz, HT‐ or UV‐NiO_x_/quartz. All the perovskite films on different surfaces possess a similar bandgap of ≈1.57 eV, but with slightly different absorbance. The perovskite film on UV‐NiO_x_/quartz has a similar absorbance to the one on quartz but slightly larger than the one on HT‐NiO_x_/quartz, so UV‐NiO_x_ has a negligible parasitic light absorption when combined with the perovskite film. Steady‐state photoluminescence (PL) and time‐resolved photoluminescence (TRPL) were also taken to probe the charge transfer kinetics between perovskite and NiO_x_ layers in the above three samples. PL quenching is regarded as an indicator to monitor the charge transfer between heterostructures.^[^ ^17^ ^]^ Figure 4a shows the PL spectra of the above three samples. Significant PL quenching is observed on both perovskite/NiO_x_/quartz samples while a much more PL quenching is found on perovskite/UV‐NiO_x_/quartz, which means a better charge transfer through the perovskite/UV‐NiO_x_ interface. To verify this point, TRPL spectra were attained as shown in Figure 4b. The carrier lifetime of perovskite layer reduces from 1956.69 to 985.43 and 797.86 ns in the presence of HT and UV‐NiO_x_, respectively. The details of TRPL value are shown in Table S2 (Supporting Information) for reference. These data support the fact that the charge carriers within perovskite layer can be extracted more effectively by UV‐NiO_x_ film than HT‐NiO_x_ film.^[^ ^7a^ ^]^ The excellent hole extraction ability contributes to the enhancement of open‐circuit voltage and fill factor in PSCs.^[^ ^5a^ ^]^ The hole‐only device was also prepared with a configuration of glass/ITO/HT or UV‐NiO_x_/perovskite/Cu to evaluate the interfacial trap density *N*_trap_. As shown in Figure 4c,d, *V*_FTL_ is set at 0.82 and 0.54 V for the perovskite on HT and UV‐NiO_x_, respectively. The perovskite films on UV‐NiO_x_ exhibit lower *N*_trap_ (5.94 × 10^15^) than that on HT‐NiO_x_ (9.02 × 10^15^).^[^ ^17^, ^18^ ^]^ This result indicates that UV‐NiO_x_ film can effectively suppress the trap sites of perovskite films.

From the above comprehensive characterizations, it is concluded that UV‐NiO_x_ film has a better hole extraction ability when compared with HT‐NiO_x_ film. This can reduce the trap density in the perovskite/NiO_x_ interface without any observable negative impacts on the morphology of as‐formed perovskite layer and interface physical contact. This conclusion is consistent with the characterization results of NiO_x_ films in Figure 3.

### 2.4 HT and UV‐NiO_x_‐Based PSCs

**Figure 5:**
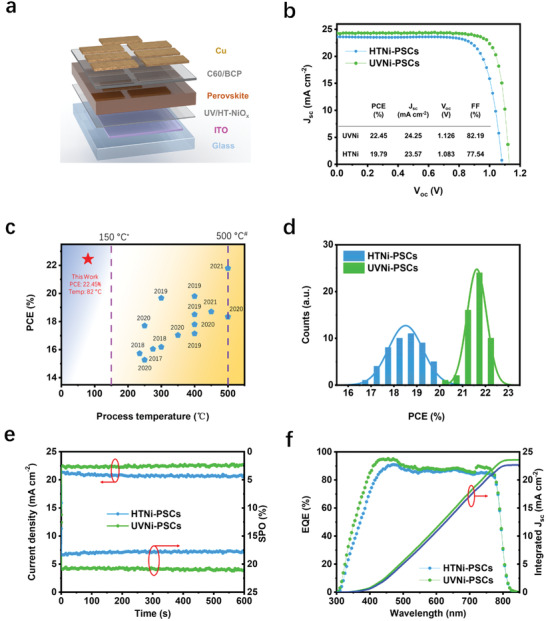
Perovskite device architecture and performance characterization. a) Schematic diagram of inverted PSCs, b) *J–V* curves of the champion devices, c) Summary of efficient solgel NiO_x_‐based inverted PSCs reported in recent years versus annealing temperature, d) statistics of PCE from 60 devices, e) SPO and f) EQE spectra of UV‐Ni‐PSCs and HTNi‐PSCs. ^*^Represents maximum processing temperature of flexible substrate (150 °C), ^#^Represents glass transition temperature (500 °C). The resistance of transparent conductive oxides, especially ITO, increases significantly at 500 °C.

Inverted PSCs with ITO/NiO_x_/CsFAMA/C_60_/BCP/Cu structure (**Figure** **5**a) were fabricated to highlight the merits of UV‐NiO_x_ for photovoltaic applications. We studied the structure and component of devices through STEM (Figure S6, Supporting Information). Functional layers were clearly distinguished from the cross‐section images with EDX mapping. The monolithic perovskite layers were observed in both HT and UV‐NiO_x_‐based devices, representing the well‐preparation of devices. The optimized conditions of 0.03 m NiO_x_ precursor and UV irradiation time of 10 min were applied in the whole work, if not specifically mentioned (Figure S7, Supporting Information).

The performances of triple cation perovskite PSCs with HT‐NiO_x_ (HTNi‐PSCs) or UV‐NiO_x_ (UVNi‐PSCs) HTLs are comprehensively investigated and analyzed. The detailed comparison of device parameters is summarized in the inserted table in Figure 5b. The champion UVNi‐PSCs has a PCE of 22.45%, which is significantly boosted compared to the champion HTNi‐PSCs (19.79%). For comparison, the results of sol–gel‐based NiO_x_ PSCs in recent years are summarized in Table S3 and Figure S8 (Supporting Information). By comparing these pioneering works, the PCE of 22.45% of our champion device is achieved by an integral contribution from admiring *V*_oc_ (1.126 V), large current density (24.25 mA cm^–2^), and a record of 82.19% for *FF*. Figure 5c shows the trend that the higher the annealing temperature is, the higher the PCE is obtained. Recently, Zhang et al. reported the sol–gel NiO_x_‐based inverted PSCs with a champion PCE of 21.79% while a process temperature as high as 500 ℃ was needed; this was quite close to the softening temperature of the glass. Our work is currently the only one to fill the gap in the upper left area of Figure 5c (PCE > 22% and *T*_process_ < 150 ℃), which has been long desired in the field of sol–gel NiO_x_‐based inverted PSCs. It also proves that the crystallization and intentional doping are not necessary for high‐performance inorganic carrier transport materials and thus the high temperature and tedious doping process can be ruled out. Also, to verify the application of UV‐NiO_x_ film preparation method in flexible devices fabrication, we prepared flexible devices; the flexible device PCE reached 19.70% (Figure S9, Supporting Information) which is listed as one of the top efficiencies of flexible inverted PSCs.^[^ ^19^ ^]^

To investigate the reproducibility of these devices, 60 pieces of HTNi and UVNi‐PSCs were fabricated, and their performances were statistically shown in Figure 5d and summarized in Table S4 (Supporting Information). The standard deviation of PCE of UVNi‐PSCs (0.3%) was smaller than that of HTNi‐PSCs (0.68%), indicating a better reproducibility. It is noted that the *FF* of UVNi‐PSCs has a superior average value of 80.89%, which is 7.39% higher than that of HTNi‐ PSCs (75.32%). In addition to high efficiency, the use of UV‐NiO_x_ HTL led to a suppressed hysteresis (Figure S10a, Supporting Information), providing more reliable PCE data derived from *J–V* curves. Further, the steady‐state output of unencapsulated HTNi and UVNi‐PSCs were examined in air (*T* = 25 °C, 60% relative humidity) at their maximum power points (MPPs) for 600 s (Figure 5e). UVNi‐PSCs delivered a stabilized *V*_oc_ of 0.96 V, *J_sc_* of 22.3 mA cm^–2^, and a corresponding PCE of 21.41%, higher than those of HTNi‐PSCs (0.86 V, 21.3 mA cm^–2^, and 18.3%). This result also indicates excellent illumination stability for UVNi‐PSCs. To verify the *J*_sc_ values from *J–V* curves, the external quantum efficiency (EQE) was conducted (Figure 5f). It was found that UVNi‐PSCs device had a higher EQE than HTNi‐PSCs device at the wavelength of 300 to 450 nm; this was consistent with the UV–vis spectrum of NiO_x_ films (Figure 2d) where the UV‐NiO_x_ film had a higher transmittance at the wavelength between 300 and 400 nm. A high transmittance donated high photocurrent generation and resulted in a high current density.^[^ ^5a^ ^]^ Finally, the stability test of UVNi and HTNi‐PSCs was conducted on all devices that were stored in ambient air (*T* = 25 °C, 60% relative humidity) as revealed in Figure S11 (Supporting Information). UVNi‐PSCs maintained 85% of initial PCE after 960 h (40 days) storage, while the HTNi‐PSCs were reduced to 81% of initial PCE. From Figure 5e and Figure S11 (Supporting Information), it was concluded that UV‐NiO_x_ film endowed PSCs excellent light and moisture stability.

To rationalize the superior performance of UVNi‐PSCs, further investigations were carried out on the interfacial traps and recombination in PSCs devices, by *I–V* measurement under various light illuminations and in the dark. The dependence of *V*_oc_ on the light intensity is thus provided in Figure S12a–c (Supporting Information). A slower photovoltage decay is observed in UVNi‐PSCs device compared to HTNi‐PSCs device; this indicates reduced recombination losses at UV‐NiO_x_/perovskite interfaces as revealed in Figure 4. Electrochemical impedance spectroscopy (EIS) in the dark with frequencies ranging from 1 MHz to 1 Hz was measured to understand the charge transfer and recombination dynamics in the devices. The fitted Nyquist plots of the EIS curves of UVNi and HTNi‐PSCs are shown in Figure S12d (Supporting Information). *R_s_* is referring to series resistance and *R*_rec_ is the recombination resistance of PSCs devices. HTNi‐PSCs device has *R*_s_ of 185.7 Ω and *R*_rec_ of 4366 Ω, whereas *R*_s_ of UVNi‐PSCs device is reduced to 59.5 Ω with the promoted *R*_rec_ to 7677 Ω. This result agrees well with the conclusion that UV‐NiO_x_ layer is in favor of improving charge transfer and suppressing the charge recombination in PSC device (Figure 2 **–** 4).^[^ ^20^ ^]^ Therefore, it is concluded that the significantly enhanced performance of UVNi‐PSCs derives is routed in the effective role of UV‐NiO_x_ film.

Further, we analyze the merits of UV irradiation method from the aspects of energy, cost, and environment. Compared to conventional annealing treatment, UV irradiation is more uniform, efficient, and thus energy‐saving.^[^ ^11^, ^21^ ^]^ In Table S5 (Supporting Information), we compare the cost of raw materials for the preparation of HTLs by UV, HT‐NiO_x_, and PTAA (a known organic HTL, efficient and cheap). It is found that for the preparation of UV‐NiO_x_ HTL, the cost can be reduced by a factor of ≈9000 (compared to PTAA) or ≈58 (compared to HT‐NiO_x_). This is in addition to the fact that the film preparation process is free of toxic organic or alcoholic solvents, and greenhouse gases. All in all, to the best of the authors’ knowledge, the proposed method in this work is the most economic and eco‐friendly method to produce doping‐free NiO_x_ HTLs for efficient inverted PSCs.

## 3 Conclusion

In this study, we present a simple, economic, eco‐friendly, and commercially applicable metal oxide synthesis method that greatly enhances the PCE of doping‐free NiO_x_ sol‐gel‐based inverted PSCs to 22.45%. Due to the merits of UV irradiation‐induced synthesis NiO_x_ method, high‐temperature annealing (>150 °C) is not required, which is a promising HTL preparation technique compatible to other existing protocols for the fabrication of flexible and tandem PSCs. Also, our method greatly reduces the cost of materials and process and is free of toxic or organic solvents and greenhouse gases, and thus represents a highly competitive strategy to prepare high‐quality inorganic hole transport layers based on the photochemical reaction kinetics. The protocol provided here is anticipated to be useful for other optoelectronic applications and commercialization.

## 4 Experimental Section

### Materials

Formamidinium iodide (FAI, 99.9%), methylammonium chloride (MACl, 99.9%), cesium iodide (CsI, 99.999%), methylammonium bromide (MABr, 99.9%), and lead iodide (PbI_2_, 99.99%) were purchased from Advanced Election Technology Co. Ltd. (P. R. China). C_60_ (99.99%) and bathocuproine (BCP, 99%) were purchased from Xi'an Polymer Light Technology Corp. (P. R. China). Nickel(II) acetylacetonate (Ni(acac)_2_, 95%), hydrochloric acid (ACS reagent, 37%), ammonium hydroxide (NH_4_OH, 28.0‐30.0% NH_3_ basis), nickel hydroxide, nickel(II) nitrate hexahydrate (Ni(NO_3_)_2_·6H_2_O, 99.999% trace metals basis), *N*,*N*‐dimethylformamide (DMF, anhydrous, 99.8%), dimethyl sulfoxide (DMSO, anhydrous, 99.9%), 2‐propanol (IPA, anhydrous, 99.5%), and ethanol (anhydrous) were obtained from Sigma–Aldrich (P. R. China). All materials were used as received.

### UV‐NiO_x_ Film Preparation

The NiO_x_ precursor solution was prepared by dissolving 0.01‐0.1 m Ni(OH)_2_ or Ni(NO_3_)_2_·6H_2_O in ammonium hydroxide to form a light blue solution, then the solution was filtered by a 0.22 µm‐PTFE filter. The precursor solution concentration of 0.03 m spun on a cleaned ITO substrate at 2000 rpm for 30–60 s, or the substrates were soaked in a diluted solution (0.005 m, pH of 14) 20–30 s and dried by N_2_ gun or spinning. Then, the substrates were transferred to a commercial UV box (250 W, Shenzhen HWO Technology Co. Ltd) for irradiation at varied time of several to tens of minutes. The film thickness was ≈13–17 nm at the concentration of 0.02 m L^−1^, 35–40 nm at the concentration of 0.05 m L^−1^, and 70–85 nm at the concentration of 0.1 m L^−1^. After the irradiation, the films were immediately transferred into the glovebox for the perovskite deposition.

### HT‐NiO_x_ Film Preparation

The HT‐NiO_x_ layer was prepared according to the previously reported method.^[^ ^6c^ ^]^ In brief, 0.1 m of Ni(acac)_2_ was dissolved in ethanol with 1% v/v HCl. The solution was filtered via 0.22 µm PTFE filter and spread on a cleaned ITO/glass substrate, then the substrate was spun at 4000 rpm for 40 s. The substrates were transferred into a N_2_ glovebox after annealing at 180 °C for 10 min and 400 °C for 45 min.

### Device Fabrication

Patterned ITO glass was cleaned by sonication in 2% Helmanex solution, acetone, EtOH, IPA, and deionized water. NiO_x_ films were prepared as described above. The Cs_0.05_FA_0.85_MA_0.1_PbI_2.91_Br_0.09_ perovskite precursor was prepared by mixing 1.5 m A‐site salt with a molar ratio of CsI:FAI:MABr = 0.05:0.85:0.1 and 1.6 m PbI_2_ in 1 mL DMF and DMSO (8:2, v/v); an extra 0.3 m MACl was added to the precursor. The perovskite precursor solution was spread on the NiO_x_ coated substrate and spun at 1000 rpm at a ramp rate of 200 rpm s^−1^ for 10 s and 4000 rpm at the ramp rate of 1000 rpm s^−1^ for 30 s; then 130 µL of ethyl acetate was quickly dripped onto the substrate in the last 5 to 10 s. The perovskite layer was then annealed at 65 °C for 5 min and 150 °C for 10 min. Devices were completed by the thermal evaporation of 40 nm C_60_, 8 nm BCP, and 100 nm Cu. The active area of the devices was 0.12 cm^2^.

### Device Performance Characterizations

Current density–voltage characterization was conducted using a Keithley 2400 source meter under AM 1.5G irradiation. Sunlight was generated using an Enlitech SS‐F7‐3A solar simulator with 300 W Xenon lamp in ambient air (humidity ≈60%). To correctly estimate the equivalent AM 1.5G irradiance level and the mismatch factor for the tested cell, the light intensity was calibrated by National Renewable Energy Laboratories (NREL)‐calibrated Si solar cell. The *J–V* curves were extracted in a range of 1.2–0.2 V with 0.2 V stepwise. The cells were measured multiple times (typically five to eight scans in 1—2 min) until reaching the performance peak. The 0.09 cm^2^ metal mask was used for *J–V* measurement. The external quantum efficiency (EQE) measurements were performed using Enlitech QE‐R3011 EQE system in ambient temperature. A 300 W Xenon lamp was used as the light source, and the light intensity was calibrated with NREL‐calibrated reference Si photodiode.

### Film Characterizations

The morphologies of both NiO_x_‐coated ITO films and perovskite films were characterized using a ZEISS Merlin SEM at an accelerating voltage of 3 kV. The Raman spectra of products and the pulse laser were obtained by using a HORIBA Raman spectrometer (LabRAM HR Evolution) with an excitation wavelength of 532 nm. The data related to the surface potential and roughness were collected using a Dimension Fastscan Atomic Force Microscope (Bruker Fastscan) in the KPFM taping mode. The SCM‐PIT‐V2 probe coated with PtIr was used for KPFM and AFM image acquisitions. The diffraction patterns were obtained using a Bruker ECO D8 diffractometer with Cu K*α* (*λ* = 1.5418 Å) radiation. UPS and XPS measurements were carried out using an ESCALAB 250Xi (Thermo Fisher Scientific) in an ultrahigh vacuum with a base pressure of 1 × 10^–10^ mbar. A monochromatic aluminum (K*α*) X‐ray source providing photons with the energy of 1486.7 eV for XPS, and a standard helium‐discharge lamp with He I*α* photons at 21.22 V for UPS. The total energy‐resolutions of XPS and UPS were 0.30 eV and 50 meV, respectively. The XPS data was analyzed and calculated using the Avantage software; the ratio of nickel components was obtained from the ratio of peaks area. The work function of the film was extracted from the edge of the secondary electron cut‐off of the UPS spectra by applying a bias voltage of −5 V to the sample. The steady‐state and time‐resolve photoluminescence spectra were collected from Edinburgh Instruments FLS 1000. Samples were prepared on a quartz for all photoluminescence measurements. The 470 nm wavelength was used for the excitation light for the steady‐state photoluminescence measurement, and a pulsed laser with 405 nm light‐emitting diode was utilized for the time‐resolve photoluminescence measurement. To understand the performance of carrier transport and hole extraction at the interface, the incident beam was emitted from the quartz side passed through the hole transport layer to excite the perovskite layer. The absorbance spectra for all films were collected using PerkinElmer lambda 950 UV–vis‐NIR spectrometer. The electrochemical impedance spectroscopy (EIS) measurements were performed on a Zahner PP211 electrochemical workstation with max‐power‐point voltage for each device. The NiO_x_ particle STEM‐DF2 images and the selected area electron diffraction patterns were obtained from FEI Talos F200X with X‐FEG gun operated at 200 kV. The preparation of particle samples was different from the film preparation. For the STEM‐DF2 images, solutions were distributed on the glass substrate without spin coating, then the substrate was transferred on a hot plate for high‐temperature annealing in the commercial UV box for photon‐induced synthesis. So, the particles had a variety of sizes for the solar cells’ fabrication with a relatively similar crystallinity. Device cross‐section TEM lamellae was prepared using Thermofisher Scientific Helios 5 UX DualBeam. Tungsten was coated on the top of the sample for protecting sample damaging from the ion beam; Ga^+^ ion beam was used for TEM lamellae preparation; the lamellae underwent a final thinning to 50–60 nm with 2 kV 43 pA to ensure a comparable gesture for STEM image and spectrum analysis. The STEM‐HAADF image with EDX mapping was obtained from FEI Titan Cubed Themis G2 double Cs‐corrected TEM with X‐FEG gun operated at 300 kV in the STEM mode. The electron beam current was reduced using Monochromator to minimize the damage in perovskite cells during the characterization. Device cross‐section EDX mapping was obtained from four Si SuperX EDX detectors.

## Conflict of Interest

C.C. and Q.L. are the inventors on a patent application related to this work filed by Southern University of Science and Technology (CN 202110220543.4). The other authors declare no competing interests.

## Supporting information

Supporting Information

## Data Availability

The data that support the findings of this study are available from the corresponding author upon reasonable request.
