# Supplementary material for: Doping Free and Amorphous NiOx Film via UV Irradiation for Efficient Inverted Perovskite Solar Cells
Source: Adv Sci (Weinh). 2022 Apr 25;9(18):2201543. doi: 10.1002/advs.202201543 (PMC9218651; doi:10.1002/advs.202201543)
Supplement: Supplementary file 1 — Supporting Information [file ADVS-9-2201543-s001.pdf]

ADVANCED SCIENCE
Open Access

Supporting Information

for *Adv. Sci.*, DOI 10.1002/advs.202201543

Doping Free and Amorphous NiO_x Film via UV Irradiation for Efficient Inverted Perovskite Solar Cells

Qing Lian, Peng-lai Wang, Guoliang Wang, Xian Zhang, Yulan Huang, Dongyang Li, Guojun Mi, Run Shi, Abbas Amini, Liang Zhang and Chun Cheng**

Supporting Information

Doping Free and Amorphous NiOx Film via UV Irradiation for Efficient Inverted Perovskite Solar Cells

Qing Lian, Peng-lai Wang, Guoliang Wang, Xian Zhang, Yulan Huang, Dongyang Li, Guojun Mi, Run Shi, Abbas Amini, Liang Zhang*, Chun Cheng*

Dr. Q. Lian, G. Wang, X. Zhang, Y. Huang, D. Li, G. Mi, Dr. R. Shi, Prof. C. Cheng

Department of Materials Science and Engineering, Southern University of Science and Technology, Shenzhen, Guangdong Province 518055, China

E-mail: chengc@sustech.edu.cn

P. Wang, Prof. L. Zhang

School of Chemistry and Molecular Engineering, East China Normal University, Shanghai 200062, China

E-mail: zhangliang@chem.ecnu.edu.cn

Prof. A. Amini

Center for Infrastructure Engineering, Western Sydney University, Kingswood, NSW 2751, Australia

Keywords: UV irradiation, photochemistry synthesis, inverted perovskite solar cells, green synthesis, NiO_x

Further Discussions

1. Raman analysis

In the XPS spectrum of HT-NiO, we can observe a peak representing Ni^{3+}, but we fail to fit out the peaks for Ni^{3+} in HT-NiO_x, the same as for the Raman Spectrum of UV-NiO_x. According to the other reports in literature, Raman peak of Ni^{3+} is not obvious for NiO_x.[1] We believe that this is because the Raman intensity of Ni^{3+} is much lower than that of Ni^{2+}, so the peaks of Ni^{2+} and Ni^{3+} cannot be observed simultaneously in Raman spectroscopy results. In contrast, we find out the peaks representing NiOO- groups in the HT- and UV-NiO_x Raman spectra that matches the XPS result, so, we consider this as a credible outcome.

2. Proposed reaction mechanisms for preparing HT/UV-NiO_x films

The morphologies, crystal structure, surface properties and compositions of the NiO_x films during the UV irradiation are studied to understand reaction process and mechanism. However, the morphologies, crystal structure and surface properties show no evident changes. We therefore do not present these mediocre results and only show the characterization results of the final products (**Fig.1-3, Supplementary Figures 2-3**). As for morphologies, a layer of NiO_x precursor film is formed after spin coating. The rough surface can be observed by SEM, and the surface morphology does not change significantly after different UV irradiation time. As for the crystal structure, we observe by XRD and TEM that NiO_x is still amorphous oxide after a long-time irradiation, as shown in **Figure 1b-d**. We believe that no crystal is formed during the irradiation process. As for the surface properties, we test the change of the contact angle of the film after different irradiation time, and the contact angle value was all about 25° without significant change.

The composition of the films changes with UV irradiation time. We used Raman to analyse the Raman shift of Ni-O bond, as shown in **Figure S4C**. The change over time represents that the Raman peak of the $\delta(Ni^{III}\text{-}O)$ and $\nu(Ni^{III}\text{-}O)$ gradually becomes stronger, which indicates the conversion of Ni^{2+} precursor to dehydrated NiOOH and Ni_2O_3. No more peaks appear with longer time; this suggests a stable composition of NiO_x films.

Based on above characterization results, we proposed reaction mechanism of NiO_x films in the following part.

The reaction process of HT-NiO_x is known from previous reports.[2] The HT-NiO_x, NiO_x was synthesised by pyrolysis and oxidation reaction of $Ni(acac)_2$ in air, where the greenhouse gas CO_2 was released as follows:

$$Ni(acac)_2(s) + O_2(g) \xrightarrow{HT=400\ ℃} aNiO{\cdot}bNi_2O_3{\cdot}cNiOOH(s) + H_2O(g) + CO_2(g) \quad (S1)$$

For the preparation of UV-NiO_x, the photon energy with the wavelength of 185 nm dominant in the UV lamp emission spectrum (**Figure S4a**) is calculated as 646.63 kJ mol^{-1}, which is high enough to decompose $Ni(OH)_2$ by breaking Ni-O and O-H bonds to form free radicals of Ni· and O· and H· (**Table S1**), the value of bond strength is obtained from CRC Handbook of Chemistry and Physics. [3]

In the UV box, ozone was formed by introducing photon energy into oxygen molecules as per the following reaction:

$$O_2 \xrightarrow{\text{Photon}} O_3 \quad (S2)$$

Raman data show that Ni $(OH)_2$ reacts with O_3 to form NiOOH and Ni_2O_3, we proposed the reaction equation as below:

$$Ni(OH)_2 + O_3 \xrightarrow{\text{Photon}} NiOOH + Ni_2O_3 + H_2O\uparrow + O_2\uparrow \quad (S3)$$

O_3 generated by the UV irradiation of O_2 in air has an extremely high oxidizing activity and thus it reacts with the free radicals of Ni· to form Ni^{3+} compositions of NiOOH and Ni_2O_3 at relatively low temperature of ~82 ℃.

Considering O_2/O_3 acted as the redox shuttle in the reaction procedure, the reaction formula can be simplified to (4):

Reaction formula (S2) + Reaction formula (S3) = Reaction formula (S4)

$$Ni(OH)_2 + O_2 \xrightarrow{Photon} aNiOOH{\cdot}\, bNi_2O_3 + H_2O\uparrow \quad (S4)$$

3. Determination of valence band maximum of NiO_x film

To determine the valence band/work function, ultraviolet photoelectron spectroscopy (UPS) is used to conduct work function measurement. The work function is determined as $W_f = h\nu - E_0$, where $h\nu$ (=21.22 eV) is the photon energy and E_0 is the binding energy value for the secondary electron cut-off.

ΔE is the energetic gap between valence band maximum (VBM) and Fermi level, where $\Delta E = VBM - E_f$. The work function $W_f = E_{VAC} - E_f$ represents the energy barrier to free space, preventing electrons at the Fermi level to escape from the solid. E_{VAC} is the vacuum level and set to zero eV. The VBM value of NiO_x film is thus determined as $VBM = -W_f + \Delta E$.

Figures and Tables

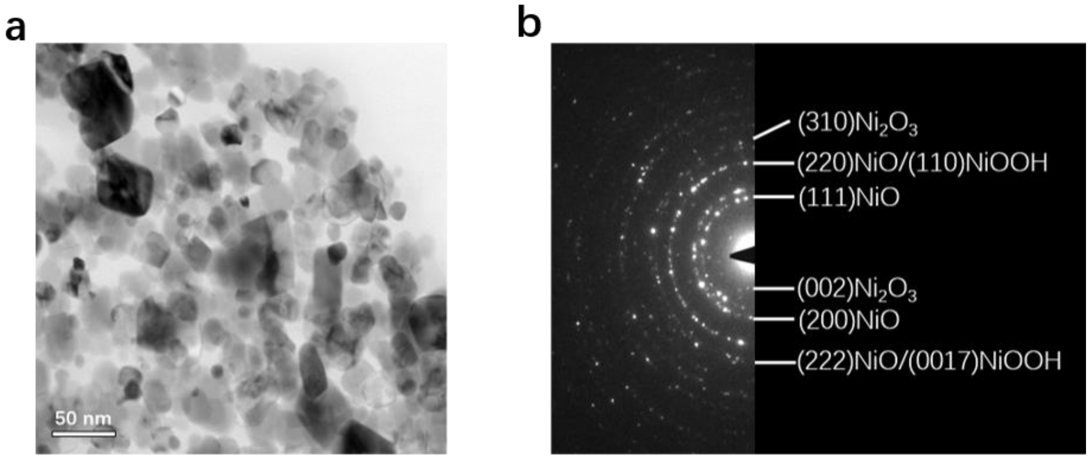

Figure S1. **Structure characterisation of HT-NiO$_x$**. (a) STEM-DF2 image, and (b) SAED pattern of HT-NiO$_x$. Scale bar is 50 nm.

Figure S1a. shows that HT-NiO$_x$ sample that is obtained by annealing at high temperature has an obvious granular shape. From **Figure S1b**, its polycrystalline structure is assessed through the electron diffraction pattern. The NiO$_x$ sample is well identified as a mixture of NiO, Ni_2O_3 and NiOOH, which is consistent with the results from the XRD characterisation (**Figure 1b**).

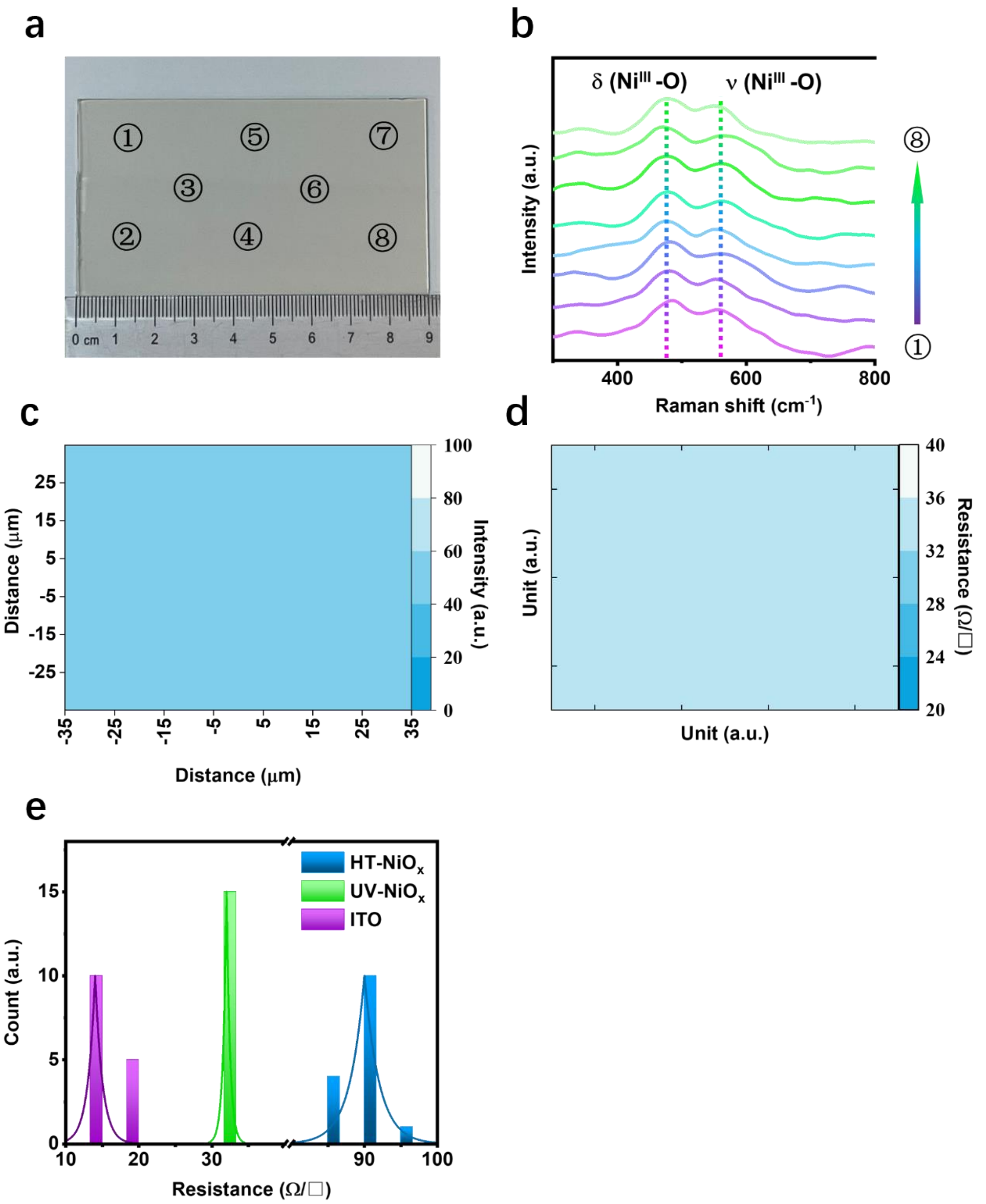

Figure S2. **NiO_x Film characterisations**. (a) Optical image of a 9 × 4.5 cm-ITO glass coated with UV-NiO_x film. (b) Raman spectra of different areas of UV-NiO_x film. (c) Raman intensity mapping of 70 × 70 μm UV-NiO_x film (step size: 7 μm in X and Y directions) at δ(Ni^{III}-O) and ν(Ni^{III}-O), (d) Resistance mapping of the large area of 9 × 4.5 cm UV-NiO_x film, (e) Resistance of HT and UV-NiO_x deposited on ITO glass.

A large area of UV-NiO_x film is prepared on 9 × 4.5 cm ITO substrate to check the uniformity of film (**Figure S2a)**. Due to the high visible light transmittance of NiO_x film, no difference is seen by naked eyes in the ITO glasses before and after NiO_x film coating. Then, the Raman spectra obtained from eight zones of the UV-NiO_x film are collected and compared (**Figure S2b)**; δ(Ni^{III}-O) and ν(Ni^{III}-O) peaks of the all selected locations are marked evenly in **Figure S2b**. The peak intensity does not show any significant change at different sites (**Figure S2b)** which indicates the good uniformity of UV-NiO_x film at large scale. The Raman mapping of a 70 × 70 μm-area further confirms the even distribution of NiO_x on ITO glass (step size 7 μm, **Figure S2c)** even at the scale of several micrometres. The sheet resistance of NiO_x films on ITO glasses is tested using the four-point probe method. The excellent conductivity of UV-NiO_x film can be well understood by knowing that Ni^{3+} donates more vacancies in the amorphous UV-NiO_x film and thus improves the electric conductivity; this has been reported previously.[4] The sheet resistance mapping of the entire UV-NiO_x film is done by four-point probe (**Figure S2d**, 6 × 8 grid), where UV-NiO_x film shows a sheet resistance distribution of 34 ± 2 Ω/□, indicating its superior uniformity in electrical conductivity. A relatively low sheet resistance with an averaged value of 32.2 ± 0.77 Ω/□ is obtained for the UV-NiO_x coated ITO glass compared to that of naked ITO glass (13.67 ± 1.18 Ω/□), while a much high sheet resistance with an averaged value of 90.53 ± 2.69 Ω/□ is obtained from HT-NiO_x coated ITO glass (**Figure S2e**, 15 sites were measured for all samples).

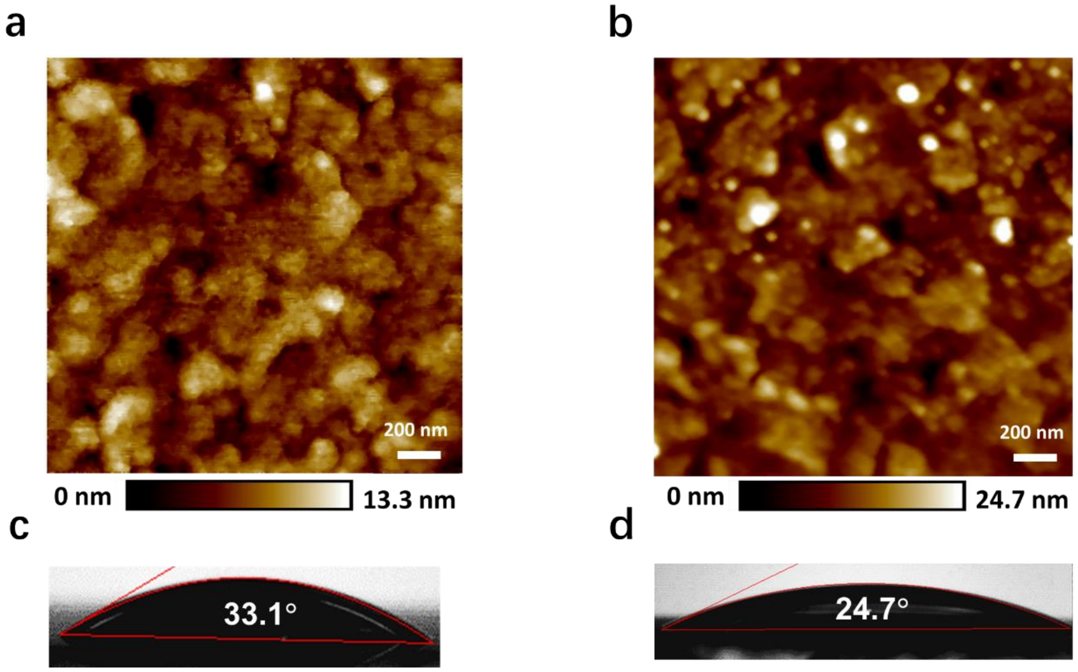

Figure S3. **NiO_x layer characterisation**. AFM images of (a) HT-NiO_x and (b) UV-NiO_x, contact angle measurement of (c) HT-NiO_x and (d) UV-NiO_x. Scale bar is 200 nm.

AFM images in **Figure S3a and b** reveal that the R_{qs} values of HT-NiO_x and UV-NiO_x films are as low as 1.84 and 3.21 nm, respectively. By combining the SEM and AFM results, it is concluded that UV-NiO_x film has a slightly rougher surface than that of HT-NiO_x film. Rougher surface may suggest better wetting property; this is supported by the results of contact angle measurement as shown in **Figure S3c and d**. A smaller contact angle of 24.7° is observed for UV-NiO_x film compared with that of HT-NiO_x film (33.1°), which suggests that perovskite precursor is more likely to spread out, favouring the formation of high-quality perovskite film on UV-NiO_x film. In addition, the improved wetting property of UV-NiO_x film is partially contributed to the fact that the UV-ozone treatment improves the surface energy via the formation of

hydroxide functional group with metal oxide.[5]

Table S1. Bond strength of NiO_x precursors. [3]

**Bond type**	**Bond strength / kJ mol^{-1}**
Ni-O	382
O-O	498
H-O	429.9
C=C	728.3
C-C	376.0

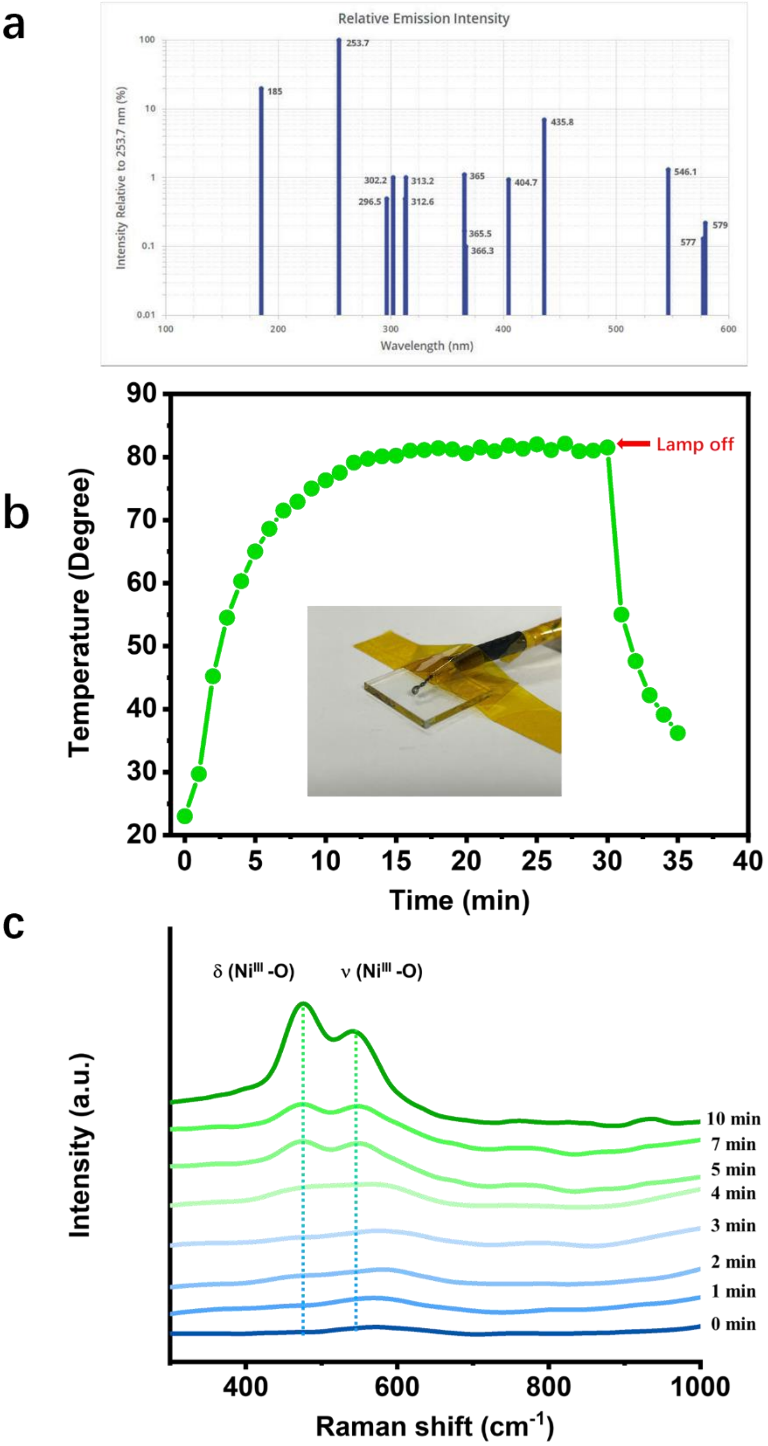

Figure S4. **Reaction conditions**. (a) UV lamp emission spectrum,[6] (b) Surface temperature as the function of irradiation time, (c) Raman spectra of UV-NiO_x film as the function of UV irradiation time.

Figure S4a shows the emission spectrum of low-pressure mercury lamp that used for UV-NiO_x synthesis. The main emission peak at 254 nm with an auditioned emission peak at 185 nm (~90% lower with respect to 254 nm). Then, we measure the variation of surface temperature with respect to irradiation time **(Figure S4b)**. The surface temperature reaches 81 ± 1 °C after 15 min irradiation, and the temperature does not further increase with the irradiation time. This result verifies that the process temperature in our protocol does not exceed 83 °C. To understand the composition of products over time, we use Raman analysis to monitor the composition of products after different UV irradiations, as shown in **Figure S4c**. The obvious peaks of $\delta(Ni^{III}$-O) and $\nu(Ni^{III}$-O) appear after 5 min UV irradiation, and the peak intensity continues increasing upon time. The result suggests that Ni^{3+} components start forming after 5 min UV irradiation.

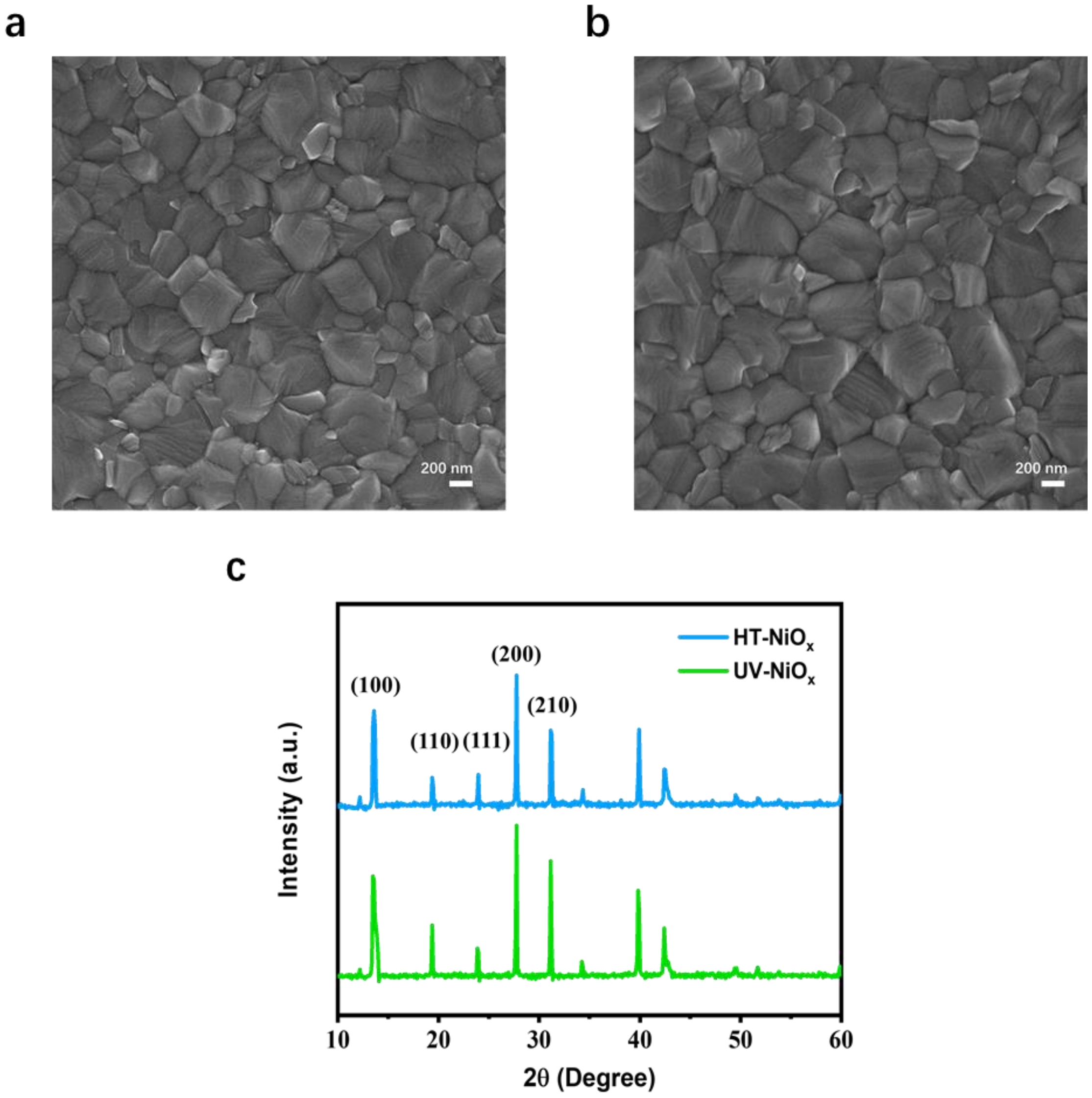

Figure S5. Perovskite layer characterisation. Top view SEM images of perovskite films prepared on (a) UV-NiO_x and (b) HT-NiO_x, (c) XRD pattern of these two perovskite films. Scale bar is 200 nm.

The top view SEM images of perovskite layer on UV-NiO_x and HT-NiO_x layers are shown in **Figure S5 a and b**, respectively. Both perovskite films with the grain size of ~400-800 nm have no pinhole, which indicates that the morphology of perovskite films on different nickel oxide films does not change significantly. Confirmed by XRD

pattern (**Figure S5c**), the perovskite structure does not have obvious changes. From SEM and XRD results, it is suggested that NiO_x films prepared by both high temperature annealing and UV irradiation methods do not affect the morphology and structure of the perovskite films.

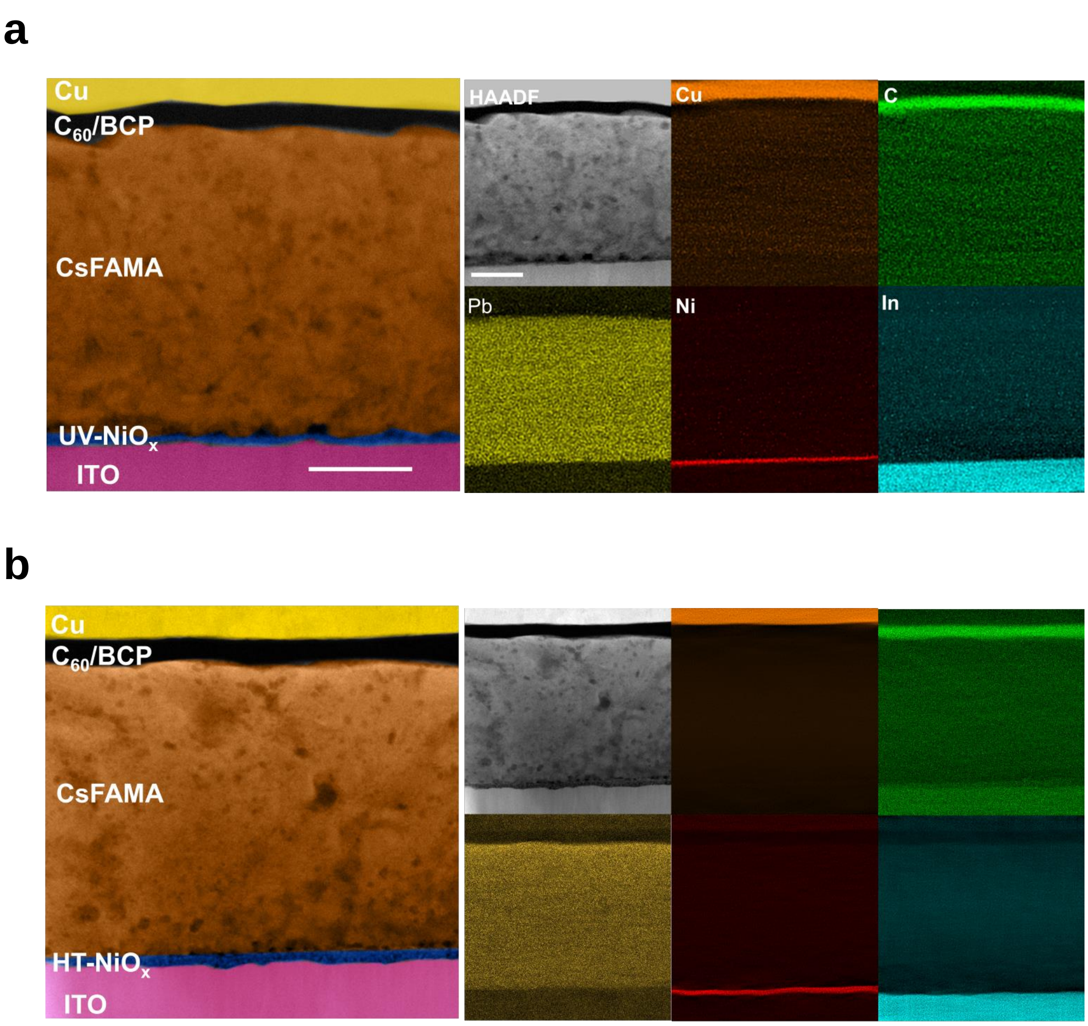

Figure S6. Cross-section STEM-HAADF images with EDX mapping of (a) UVNi-PSCs and (b) HTNi-PSCs, depicting the devices' architectures. Scale bar is 200 nm.

Cross section STEM images with the EDX mapping of PSCs devices are shown in **Figure S6a and b**. The device layers are clearly distinguished including ITO layer, NiO_x layer with the thickness of around 20 nm, monolithic perovskite layer with the thickness of around 600 nm, C_{60}/BCP layer with the thickness of around 50 nm and Cu electrodes. The above results confirm that both UVNi and HTNi-PSCs devices are well prepared.

Table S2. PL lifetime parameters by fitting TRPL curves with the double-exponential decay model.

**Sample**	**A_1 / %**	**τ_1 / ns**	**A_2 / %**	**τ_2 / ns**	**Weighted average τ / ns**
Perovskite	1.78	249.97	98.22	1960.63	1956.69
HT-NiO/ perovskite	1.50	37.99	98.50	985.99	985.43
**UV-NiO/ perovskite**	**2.74**	**39.28**	**97.26**	**798.91**	**797.86**

TRPL is used to study the efficiency of charge extraction. The TRPL spectra are tested and fitted with a biexponential function. The TRPL are calculated by fitting the curve with the following equation (S5).[7]

$$I(t) = A_1\left(-\frac{t}{\tau_1}\right) + A_2\left(-\frac{t}{\tau_2}\right) \qquad \text{(S5)}$$

And, the $\tau_{average}$ is calculated from equation S8.

$$\tau_{average} = \frac{A_1\times\tau_1^2+A_2\times\tau_2^2}{A_1\times\tau_1+A_2\times\tau_2} \qquad \text{(S6)}$$

The photoluminescence lifetime of perovskite layer varies from 1956.69 ns to 985.43 ns (for HT-NiO_x) and 797.86 ns (UV-NiO_x), which means that the charge carriers within the perovskite layer can be more effectively extracted by the UV-NiO_x layer than HT-NiO_x layer.

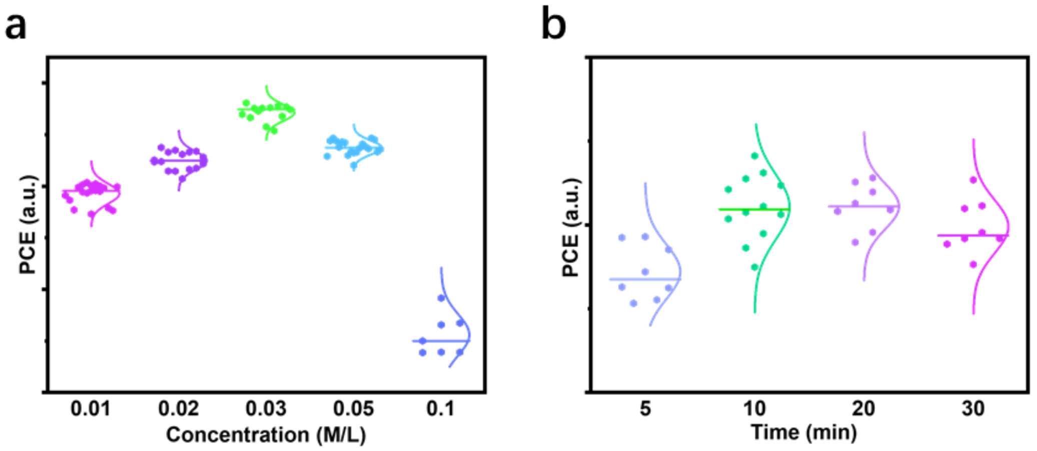

Figure S7. The PCE of UVNi-PSCs *vs.* UV-NiO_x films prepared at different (a) precursor concentrations and (b) UV-illumination times.

According to **Figure S7a**, the highest efficiency of PSCs device was obtained when the UV-NiO_x film was prepared at the precursor concentration of 0.03 M/L. **Figure S7b** shows that the efficiency of PSCs device reached the maximum when the UV-NiO_x film was prepared under the UV irradiation for 10 minutes. So, the most efficient UV-NiO_x film was prepared at the precursor concentration of 0.03 M/L and the irradiation time of 10 minutes; We used these conditions for the fabrication of the champion device, as shown in **Figure 5b**.

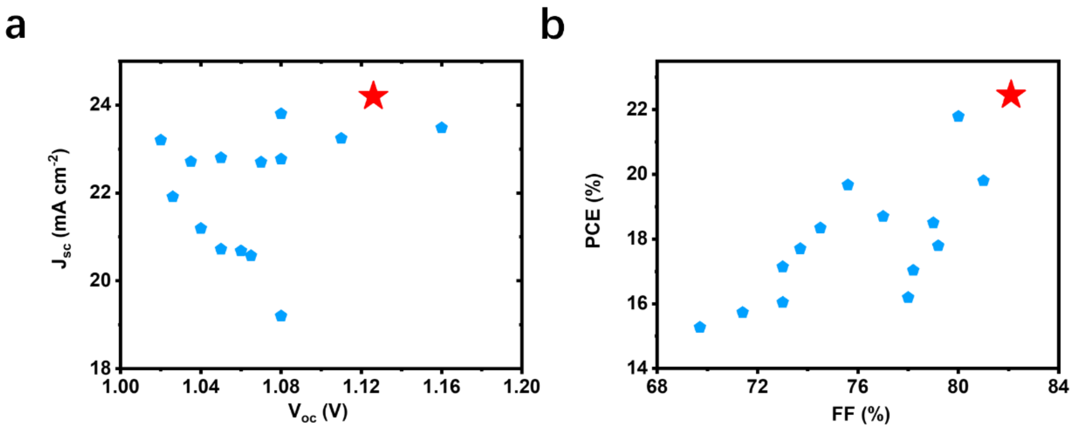

Figure S8. **Recently reported highly efficient sol-gel NiO_x-based inverted PSCs**. (a) J_{sc} *vs.* V_{oc} and (b) PCE *vs.* FF

We compared the performance of devices reported in recent years on sol-gel based HTNi-PSCs with that of our sol-gel based UVNi-PSCs (see **Table 1** for reference). Our device had a relatively high V_{oc}, J_{sc} and a record *FF*, which was among the top PCEs for sol-gel based NiO_x-PSCs.

Table S3. Summary of efficient sol-gel NiO_x-based inverted PSCs reported in recent year as per their processing temperature for NiO_x preparation.

Perovskite type	*Temperature* ℃	*PCE* %	V_{oc} V	*FF* %	J_{sc} $mA\ cm^{-2}$	Year
$FA_{0.15}MA_{0.85}Pb(I_{0.95}Br_{0.05})_3$	235(SG)	15.73	1.04	71.4	21.19	2018[8]
$MAPbI_3$	250(SG)	15.27	1.065	69.7	20.57	2020[9]
$MA_{1-x}FA_xPbI_{3-y}Cl_y$	250(SG)	17.7	1.07	73.7	22.7	2020[10]
$MAPbI_3$	275(SG)	16.04	1.06	73	20.68	2017[11]
$MAPbI_3$	300(SG)	16.19	1.08	78	19.2	2018[12]
$MAPbI_3$	300(SG)	19.67	1.11	75.6	23.24	2019[5]
$MAPbI_3$	350(SG)	17.03	1.05	78.2	20.72	2020[13]
$MAPbI_3$	400(SG)	17.14	1.035	73	22.71	2019[14]
$Cs_{0.05}MA_{0.16}FA_{0.79}Pb_{1.03}(Br_{0.16}I_{0.86})_3$	400(SG)	17.79	1.026	79.2	21.91	2020[15]
$(FA_{0.83}MA_{0.17})_{0.95}Cs_{0.05}Pb(I_{0.9}Br_{0.1})_3$ ($BMIMBF_4$)	400(SG)	19.8	1.08	81	23.8	2019[2a]
$(FA_{0.83}MA_{0.17})_{0.95}Cs_{0.05}Pb(I_{0.9}Br_{0.1})_3$	400(SG)	18.5	1.02	79	23.2	2019[2a]
$Cs_{0.05}(MA_{0.15}FA_{0.85})_{0.95}Pb(Br_{0.15}I_{0.85})_3$	450(SG)	18.7	1.05	77	22.8	2021[16]
$MAPbI_3$	500(SG)	18.34	1.08	74.5	22.77	2020[17]
$Cs_{0.05}(FA_{0.85}MA_{0.15})_{0.95}Pb(I_{0.85}Br_{0.15})_3$	500(SP)	21.79	1.16	80	23.48	2021[2b]
**$Cs_{0.05}FA_{0.85}MA_{0.1}PbI_{2.91}Br_{0.09}$**	**82(SG)**	**22.45**	**1.126**	**82.19**	**24.25**	**This work**
**$Cs_{0.05}FA_{0.85}MA_{0.1}PbI_{2.91}Br_{0.09}$ (Flexible)**	**82(SG)**	**19.70**	**1.112**	**79.57**	**22.26**	**This work**

SG: sol-gel method, SP: spray pyrolysis on FTO. * the maximum processing temperature is less than 83°C. Note: NiO_x nanocrystals based PSCs have reached a high PCE of 22.7%, but the complex procedure and 270 ° C processing temperature for the synthesis of nanocrystal as well as the dispersion issue, offset its advantage on the PCEs record when compared to the

sol-gel strategy[18].

Table S4. Devices' parameters for UVNi and HTNi-PSCs.

**Device**	**PCE (%)**	**V_{oc} (V)**	**FF (%)**	**J_{sc} (mA cm^{-2})**	**J_{sc} (EQE) (mA cm^{-2})**
HTNi-PSCs	18.89 ± 0.68	1.076 ± 0.007	75.32 ± 2.08	23.31 ± 0.24	-
Champion	19.79	1.083	77.54	23.57	22.66
UVNi-PSCs	21.66 ± 0.30	1.118 ± 0.007	80.89 ± 1.05	23.94 ± 0.25	-
**Champion**	**22.45**	**1.126**	**82.19**	**24.25**	**23.58**

We studied 60 devices for UVNi and HTNi-PSCs. It was found that the PCE parameters of devices based on UV-NiO_x were all higher than those of devices prepared on HT-NiO_x. Specifically, the FF of device was significantly improved by using UV-NiO_x with an average FF increased from 77.54% for HTNi-PSCs to 80.89% for UVNi-PSCs. In addition, the standard deviation of PCE of UVNi-PSCs was smaller than that of HTNi-PSCs, indicating better reproduceability.

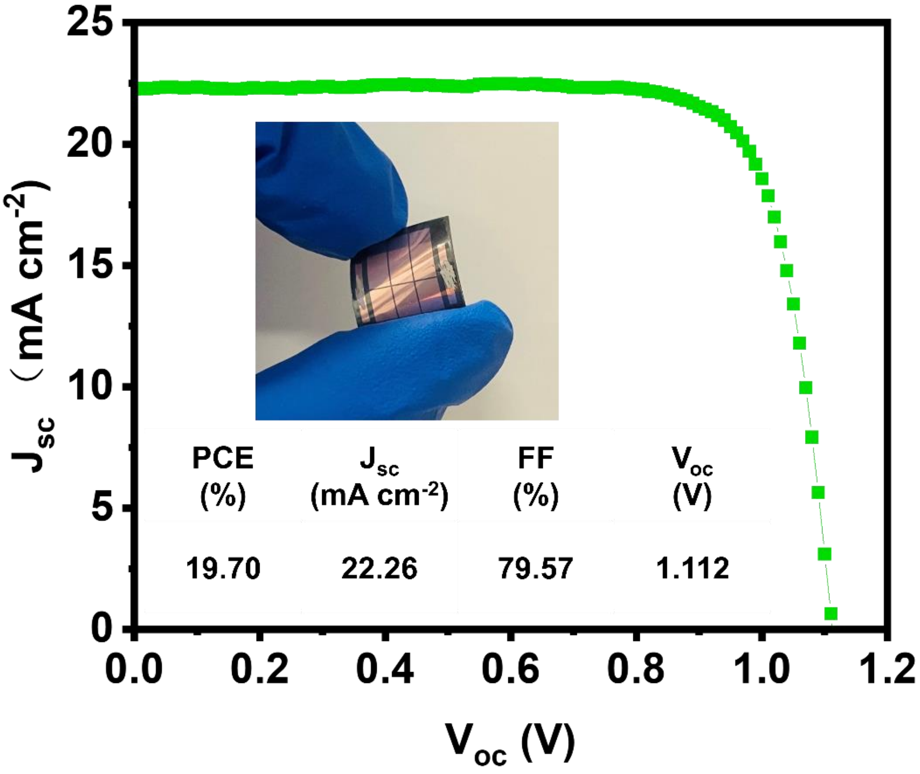

Figure S9. **Flexible device performance**. J-V curve of reverse scan of UVNi-PSCs based on PEN flexible substrate.

We fabricated flexible UV-NiO_x based perovskite solar cells. The champion device shows the performance as high as 19.70%.

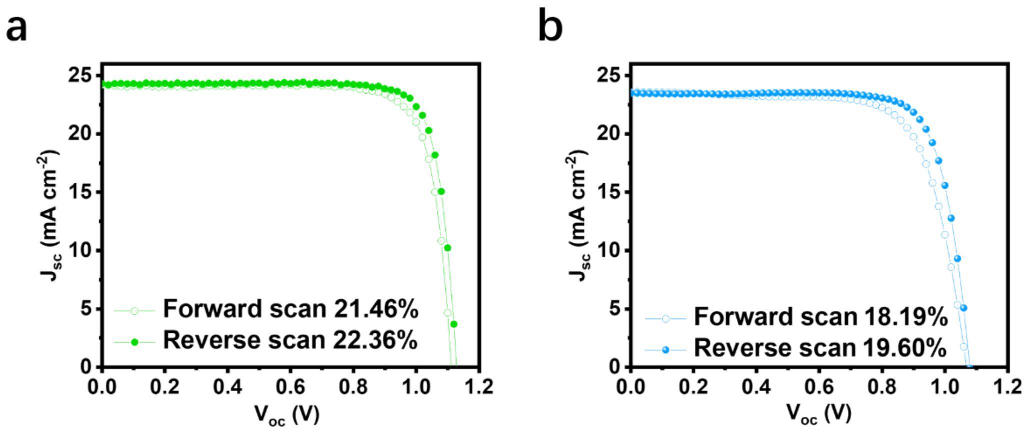

Figure S10. **Characterisation of devices' hysteresis**. Forward and reverse scans of (a) UVNi-PSCs and (b) HTNi-PSCs.

It was also found that UVNi-PSCs had a lower hysteresis than that of HTNi-PSCs. This result verified UVNi-PSCs with a better stability than HTNi-PSCs.

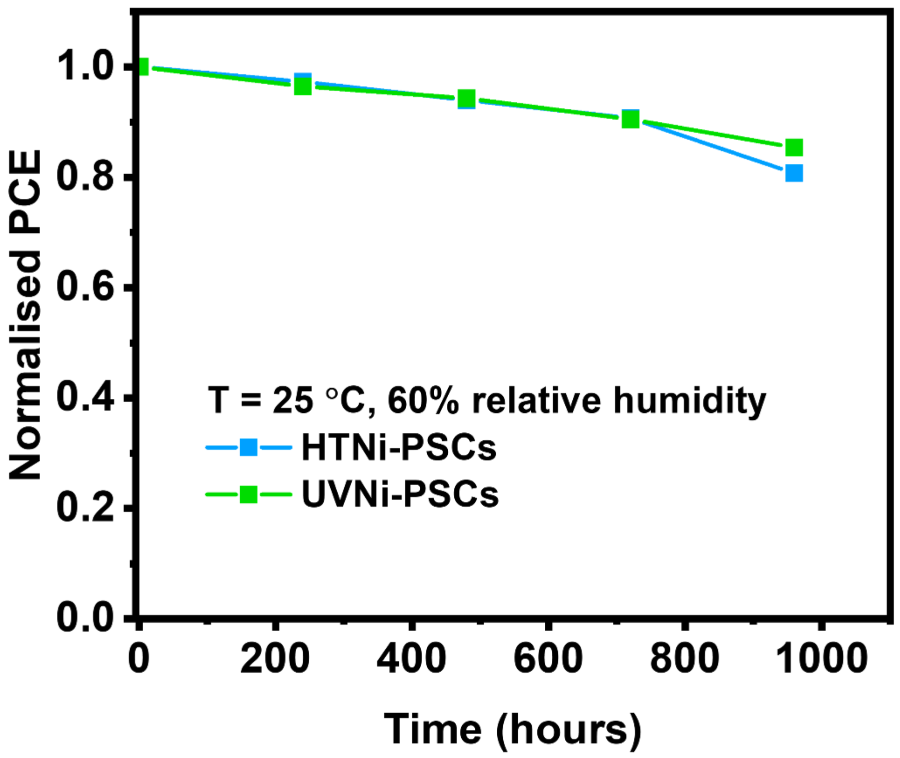

Figure S11. Long term stability test of the optimal UVNi-PSCs and HTNi-PSCs.

The stability test was conducted on UVNi-PSCs and HTNi-PSCs. All devices were stored in ambient air (T = 25 °C) without encapsulation; their stability results are shown in **Figure S11**. The UVNi-PSCs maintained 85% of initial PCE after 960 hours (40 days) storage in air, while PCE of HTNi-PSCs was reduced to 81% of initial PCE. These results showed that UV-NiO_x layer could slightly improve the stability of PSCs.

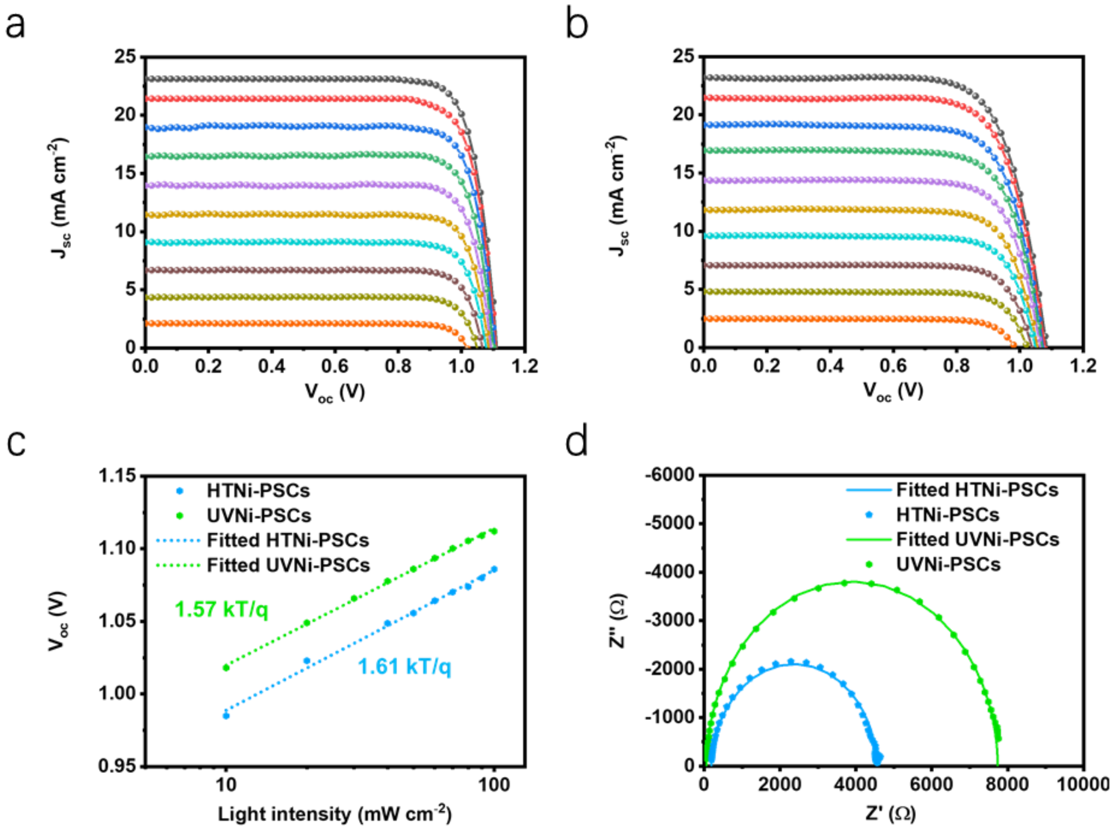

Figure S12. J-V curves of (a) UVNi-PSCs and (b) HTNi-PSCs under different illumination intensities, (c) evaluation of V_{oc} as a function of light illumination intensity, (d) Nyquist plots of EIS curves of UVNi-PSCs and HTNi-PSCs.

The dependence of V_{oc} on light intensity is given in **Figure S12c**, extracted from the light intensity dependent J-V curves in **Supplementary Figure 12a and b**. From **Figure S10c**, a slower photovoltage decay is observed in UVNi-PSCs (1.57 kT/q) compared to HTNi-PSCs (1.61 kT/q), which is coherent with the reduced recombination losses at the interfaces of UV-NiO_x/perovskite (**Figure 4 a and b**). Electrochemical impedance spectroscopy (EIS) in the dark with the frequencies ranging from 1MHz to 1Hz is used to explore the charge transfer and recombination dynamics in the devices. The fitted Nyquist plots of EIS curves of UVNi-PSCs and HTNi-PSCs are shown in **Figure**

S12d. The HTNi-PSCs device gives R_s of 185.7 Ω and R_{rec} of 4366 Ω, whereas R_s of UVNi-PSCs device is reduced to 59.5 Ω and R_{rec} is promoted to 7677 Ω. This result agrees with the fact that UV-NiO_x layer is in the favour of improving the charge transfer and suppressing the charge recombination in the device (see **Figure 4 a and b**).

Table S5. Cost analysis of the HTL layer fabrication methodologies.

**HTL**	**Raw material Price ($ g^{-1})**	**Price per m^2 ($ m^{-2})**	**PCE (%)**	**Solution base**	**Temperature**	**Annealing Time**	**Greenhouse gas (g M^{-1})**
PTAA[19]	$2,200	$275.000	23.8%[20]	Chlorobenzene	100 °C	10 min	N/A
HT-NiO_x	$4.38	$1.628	19.8%[2a]	Alcoholic	>250 °C	> 45 min	220 (CO_2)
**UV-NiO_x**	**$0.207**	**$0.028**	**22.45 %**	**Aqueous**	**RT**	**5 ~ 20 min**	**N/A**

In order to understand the economics of UV-NiO_x, we used materials density and volume to calculate the cost for NiO_x films fabrication. The thickness of NiO_x layers were about 20 nm from the cross-section STEM image of PSCs. We then estimated a volume of 0.02 cm^3 for 1 m^2 of NiO_x film. We calculated the amount of raw material used per square meter based on the nickel compounds ratio obtained by XPS and the density of various nickel compounds. The density was obtained from the Handbook of Chemistry and Physics, NiO: 6.72 g cm^{-3}, Ni_2O_3: 4.83 g cm^{-3}, NiOOH: 4.68 g cm^{-3}. Due to the use of soaking method for preparation of UV-NiO_x film, nickel oxide precursor film was coated on both sides of ITO glass, so we doubled the volume of NiO_x film in the raw materials calculation. For the preparation of HT-NiO_x, we assumed a blade coating technique was applied without any waste of precursor solution. The

preparation method of PTAA layer was referred to the methods reported in literature, each square centimeter of film requires 2.5 uL of 5 mg mL^{-1} of PTAA solution[19].

The raw material prices are referenced from Sigma Alrdich (United State) with the maximum pack size (PTAA: 1g package \$2,200 g^{-1}, nickel (II) acetylacetonate nickel (II): 50g package \$4.38 g^{-1}, nitrate hexahydrate: 500g package \$1.256 g^{-1} or nickel(II) hydroxide: 1KG package \$0.207 g^{-1}). The estimated price for raw materials per m^2 is \$275.00 for PTAA, \$1.628 for HT-NiO_x, and \$0.028 for UV-NiO_x. The state-of-the-art of the fabrication process of UV-NiO_x HTL is that no toxic organic or alcoholic solvent is used during the whole process, and the film can be well prepared within half an hour without further high temperature annealing or other post-treatments. More importantly, our method does not produce any greenhouse gases during the reaction procedure, while 220 g (5 M) of greenhouse gas per 1 M Ni raw materials is generated from the HT annealing method (**Supplementary equation (1)**). Compared to HT annealing method, UV irradiation method is an eco-friendly method, capable for mass fabrication. In short, our method is clean, simple, economic, and easy to scale-up for mass production. It is then highly recommended to replace the traditional methods with the present inorganic HTL for the commercial production of efficient PSCs.

Reference

[1] a) O. Diaz-Morales, D. Ferrus-Suspedra, M. T. M. Koper, *Chem. Sci.* **2016**, 7, 2639; b) J. Huang, Y. Li, Y. Zhang, G. Rao, C. Wu, Y. Hu, X. Wang, R. Lu, Y. Li, J. Xiong, *Angew Chem Int Ed Engl* **2019**, 58, 17458.

[2] a) S. Bai, P. Da, C. Li, Z. Wang, Z. Yuan, F. Fu, M. Kawecki, X. Liu, N. Sakai, J. T.-W. Wang, S. Huettner, S. Buecheler, M. Fahlman, F. Gao, H. J. Snaith, *Nature* **2019**, 571, 245; b) H. Zhang, K. Li, M. Sun, F. Wang, H. Wang, A. K. Y. Jen, *Adv. Energy Mater.* **2021**, 11, 2102281.

[3] J. R. Rumble, Ed.;, *CRC Handbook of Chemistry and Physics*.

[4] a) L. M. Manceriu, P. Colson, A. Maho, G. Eppe, N. D. Nguyen, C. Labrugere, A. Rougier, R. Cloots, C. Henrist, *J. Phys. D: Appl. Phys.* **2017**, 50, 225501; b) D. Di Girolamo, F. Di Giacomo, F. Matteocci, A. G. Marrani, D. Dini, A. Abate, *Chem. Sci.* **2020**, 11, 7746; c) D. Di Girolamo, M. Piccinni, F. Matteocci, A. G. Marrani, R. Zanoni, D. Dini, *Electrochim. Acta* **2019**, 319, 175.

[5] T. Wang, D. Ding, H. Zheng, X. Wang, J. Wang, H. Liu, W. Shen, *Sol. RRL* **2019**, 3, 1900045.

[6] (Ed: L. Shenzhen HWO technology Co.), Introduction of UV Cleaner.

[7] P. Ru, E. Bi, Y. Zhang, Y. Wang, W. Kong, Y. Sha, W. Tang, P. Zhang, Y. Wu, W. Chen, X. Yang, H. Chen, L. Han, *Adv. Energy Mater.* **2020**, 10, 1903487.

[8] J. Zhang, W. Mao, X. Hou, J. Duan, J. Zhou, S. Huang, W. Ou-Yang, X. Zhang, Z. Sun, X. Chen, *Sol. Energy* **2018**, 174, 1133.

[9] W. Han, G. Ren, Z. Li, M. Dong, C. Liu, W. Guo, *J. Energy Chem.* **2020**, 46, 202.

[10] B. Zhang, J. Su, X. Guo, L. Zhou, Z. Lin, L. Feng, J. Zhang, J. Chang, Y. Hao, *Adv. Sci.* **2020**, 7, 1903044.

[11] W. Chen, F.-Z. Liu, X.-Y. Feng, A. B. Djurišić, W. K. Chan, Z.-B. He, *Adv. Energy Mater.* **2017**, 7, 1700722.

[12] X. Xia, Y. Jiang, Q. Wan, X. Wang, L. Wang, F. Li, *ACS Appl. Mater. Interfaces* **2018**, 10, 44501.

[13] A. Wang, Z. Cao, J. Wang, S. Wang, C. Li, N. Li, L. Xie, Y. Xiang, T.

Li, X. Niu, L. Ding, F. Hao, *J. Energy Chem.* **2020**, 48, 426.
[14] Y. Hou, L. J. Tang, H. W. Qiao, Z. R. Zhou, Y. L. Zhong, L. R. Zheng, M. J. Chen, S. Yang, H. G. Yang, *J. Mater. Chem. A* **2019**, 7, 20905.
[15] C. C. Boyd, R. C. Shallcross, T. Moot, R. Kerner, L. Bertoluzzi, A. Onno, S. Kavadiya, C. Chosy, E. J. Wolf, J. Werner, J. A. Raiford, C. de Paula, A. F. Palmstrom, Z. J. Yu, J. J. Berry, S. F. Bent, Z. C. Holman, J. M. Luther, E. L. Ratcliff, N. R. Armstrong, M. D. McGehee, *Joule* **2020**, 4, 1759.
[16] F. Sadegh, S. Akin, M. Moghadam, R. Keshavarzi, V. Mirkhani, M. A. Ruiz‐Preciado, E. Akman, H. Zhang, M. Amini, S. Tangestaninejad, I. Mohammadpoor‐Baltork, M. Graetzel, A. Hagfeldt, W. Tress, *Adv. Funct. Mater.* **2021**, 31, 2102237.
[17] C. Hu, Y. Bai, S. Xiao, K. Tao, W. K. Ng, K. S. Wong, S. H. Cheung, S. K. So, Q. Chen, S. Yang, *Sol. RRL* **2020**, 4, 2000270.
[18] Q. Cao, J. Yang, T. Wang, Y. Li, X. Pu, J. Zhao, Y. Zhang, H. Zhou, X. Li, X. Li, *Energy Environ. Sci.* **2021**, 14, 5406.
[19] Y. Deng, X. Zheng, Y. Bai, Q. Wang, J. Zhao, J. Huang, *Nat. Energy* **2018**, 3, 560.
[20] S. Chen, X. Dai, S. Xu, H. Jiao, L. Zhao, J. Huang, *Science* **2021**, 373, 902.
